# Utility and effectiveness of Symbicort® Turbuhaler® (oral inhalation containing budesonide and formoterol) in a patient with severe asthma after permanent tracheostomy

**DOI:** 10.1186/s40780-018-0118-y

**Published:** 2018-09-10

**Authors:** Kohhei Maeda, Masao Yamaguchi, Hiroyuki Nagase, Nobuhiro Yasuno, Fumio Itagaki, Machiko Watanabe

**Affiliations:** 1 Department of Pharmacy, Teikyo University Hospital, 2-11-1 Kaga, Itabashi-ku, Tokyo, 173-8605 Japan; 2 Department of Internal Medicine Division of Respiratory Medicine and Allergology, Teikyo University School of Medicine, 2-11-1 Kaga, Itabashi-ku, Tokyo, 173-8605 Japan; 3 Faculty of Pharma Sciences, Teikyo University, 2-11-1 Kaga, Itabashi-ku, Tokyo, 173-8605 Japan

**Keywords:** Severe asthma, Dry-powder inhaler, Permanent tracheostomy, SMART, Inhalation guidance

## Abstract

**Background:**

The utility and effectiveness of inhalational asthma therapy in patients with a permanent tracheostomy has not been established. Previously, a few studies reported the use of nebulizer-type inhalers for treating these patients. Symbicort® Turbuhaler® (Symbicort) is an orally inhaled dry powder containing the corticosteroid budesonide and the bronchodilator formoterol. There are no reports describing the successful use of Symbicort in patients with a permanent tracheostomy.

**Case presentation:**

We describe the case of a woman with poorly controlled severe asthma after a permanent tracheostomy. She had developed thyroid cancer with tracheal invasion for which right thyroid lobectomy and tracheal and esophageal resection were performed, with subsequent construction of a permanent tracheostomy. In our case, prior to surgery, asthma control had been improved by adding a bronchodilator—the long-acting muscarinic antagonist tiotropium—and the anti-IgE antibody agent omalizumab to single maintenance and reliever therapy (SMART) using Symbicort; surgery was then performed. After surgery, asthma control worsened as a result of a change from Symbicort to budesonide nebulizer and a tulobuterol patch. In order to resume SMART therapy, an In-Check® inspiratory flow meter was used to measure and assess whether the inspiratory flow rate was sufficient for a dry-powder inhaler. Inhalation guidance was provided. On inhalation with the tracheostomy closed at the same time, the inspiratory flow rate was 43 L/min at the maximum. This was judged to be sufficient for the effect of Symbicort, and thus the inhaler was changed to Symbicort. Asthma symptoms promptly improved, and the patient was subsequently discharged.

**Conclusions:**

The use of Symbicort resulted in improved asthma control in a patient with severe asthma following a permanent tracheostomy. Thus, it is suggested that inhalation powder could be an option for patients with permanent tracheostomy.

## Background

Symbicort® Turbuhaler® (Symbicort), an oral inhaler containing a long-acting β2 agonist (LABA) and an inhaled corticosteroid (ICS), is prescribed to adult patients with bronchial asthma and chronic obstructive pulmonary disease. The primary components of Symbicort are the bronchodilator formoterol (LABA), which is powerful, long-lasting, and fast-acting, and budesonide (ICS), which is a highly soluble adrenocorticosteroid. In contrast to conventional therapy with a fixed amount of drug for the long-term control of asthma (mainly ICS) combined with short-acting β2 agonist (SABA) for managing occasional attacks, single maintenance and reliever therapy (SMART) with Symbicort containing ICS/LABA is a novel treatment that enables the long-term management of asthma and occasional treatment for attacks.

We encountered a patient with severe and unmanageable asthma who underwent surgery including a permanent tracheostomy for thyroid cancer involving tracheal infiltration. The efficacy of inhaled drugs in patients with permanent tracheostomy has not been established. Although case studies and small-scale studies have investigated their efficacy, nebulizer-type drugs have been the most frequently analyzed [1, 2]. Here, we report this case in which asthma symptoms were improved after switching therapy from nebulizer-type inhaled medication to Symbicort inhalation powder, which is available only as a dry-powder inhaler (DPI) formulation in Japan.

## Case presentation

Patient: A 76-year-old woman.

Chief complaint: To improve asthma management before undergoing neck surgery.

Past medical history: Diabetes, hypertension, pollen allergy.

Tobacco use: No.

Current medication (antiasthmatic drugs): Symbicort® Turbuhaler® (Symbicort), 2 inhalations twice daily up to a total of 8 inhalations per day (SMART); montelukast tablets 10 mg/day; theophylline sustained-release tablets 400 mg/day; ketotifen capsules 2 mg/day; salbutamol inhalation 0.5%, 0.5 mL/asthma attack; and prednisolone tablets 5 mg, to be taken at the patient’s discretion at the time of an attack.

Respiratory function test: Forced vital capacity, 2.74 L (129.2%); forced expiratory volume for 1 s (FEV1), 1.09 L (76.8%); FEV1, 39.8%, with findings of obstructive ventilatory defect.

Current medical history: Adult-onset asthma. Despite undergoing Step 4–5 (Global Initiative for Asthma 2017 [GINA2017]) therapy as a long-term management approach, wheezing persisted, and she had been admitted to a nearby hospital once every 2 years or so due to asthma attacks triggered by irregular weather conditions.

She was referred by a nearby otolaryngologic clinic to the Department of Otorhinolaryngology in our hospital for thorough examination of hoarseness lasting for 1–2 years. Examination findings revealed thyroid cancer with tracheal infiltration for which radical surgery was indicated. However, because wheezing increases surgical risk, she was referred to the Department of Internal Medicine for preoperative control of intractable wheezing. Consistent with her complaint of constant wheezing, initial examination revealed expiratory wheezing at rest.

To improve asthma management during a 4-week period before surgery, we prescribed Spiriva® Respimat® (Spiriva Respimat) 2.5 μg (two inhalations once daily) and subcutaneous injections of 300 mg anti-IgE antibody omalizumab every 2 weeks (determined based on the level of serum IgE [159 IU/mL IgE specific to *Aspergillus fumigatus*] and body weight [50.9 kg]).

Owing to these additions, wheezing disappeared after 1 week, and so the patient was able to undergo right thyroid lobectomy with tracheoesophageal resection as scheduled.

Considering that it would be difficult for the patient to use the inhalation powder Symbicort because of a permanent tracheostomy postoperatively, we switched from Symbicort to nebulized budesonide 2000 μg/day and a tulobuterol patch (2 mg). Spiriva Respimat and omalizumab were continued after surgery.

Approximately 3 weeks after surgery, the patient began to develop respiratory distress and wheezing early in the morning. According to the patient, these attacks would have been managed quickly as small attacks by additional inhalations of Symbicort, suggesting that the nebulized SABA that she had been taking postoperatively was not sufficiently effective. Because these symptoms used to be well managed when Symbicort was used, we considered switching back to Symbicort from nebulized budesonide and the tulobuterol patch. In Japan, however, Symbicort is available in only a DPI formulation, Turbuhaler®. In order to maximize the beneficial effects of Symbicort Turbuhaler, inspiratory flow rates need to exceed a certain level, but our patient would likely have had insufficient inspiratory flow rate due to the tracheostomy, and would thus need a device that does not require a high inspiratory flow rate, such as a pressurized metered-dose inhaler (pMDI). Adoair® and Flutiform® aerosols are commercially available pMDIs in Japan and contain ICS/LABA. Flutiform® aerosol was considered the most likely candidate because it contains the same LABA (formoterol), but not ICS, as in Symbicort, but Flutiform may leak out from the tracheostomy after being sprayed intraorally. Because the patient had previously been undergoing SMART with additional inhalations of Symbicort for acute exacerbations, it would be best to use Symbicort if possible. There seems to be no difference in the degree of distribution to the respiratory tract whether a tracheostomy is present or not if the patient is able to close the tracheostomy using his or her hand. However, the drug distribution in the respiratory tract is unknown when a patient with a tracheostomy uses an inhaled drug. Since the diameter of the tracheostomy is about 1 to 2 cm, the dead space can potentially be ignored. When the effect was found to be inadequate, we considered a change to Flutiform®, which has similar components in a different device (pMDI), because the tracheostomy was just under the skin in this patient.

Two types of devices are used to measure inspiratory flow rate: the whistle-type Turbutester (accessory for the Turbuhaler®) and the In-Check Dial®, an inspiratory flow measurement device. The Turbutester generates an audible whistling sound during inhalation, signifying that the patient has an adequate inspiratory flow rate to use Symbicort. The In-Check Dial, when used with an adapter tailored to conventional inhalers, functions as an inspiratory flow measurement device and expresses inspiratory flow rates in L/min. Our patient had an inspiratory flow rate barely sufficient to generate a whistling sound from the Turbutester during inhalation while simultaneously closing the tracheostomy with her hands. So, to select the best inspiratory flow rate, she was instructed to measure the rate three times using the In-Check Dial®, the Turbuhaler®, and the special adaptor while closing tracheostomy. The results were 40, 43, and 43 L/min. Several inspiratory flow rates have been suggested to be required to exert the beneficial effect of Turbuhaler®, but the consensus is that inspiratory flow rates above 30 L/min are sufficient to use Turbuhaler® [3, 4]. These findings, together with the findings from the Turbutester, suggest that the present patient could benefit from these devices.

**Fig. 1:**
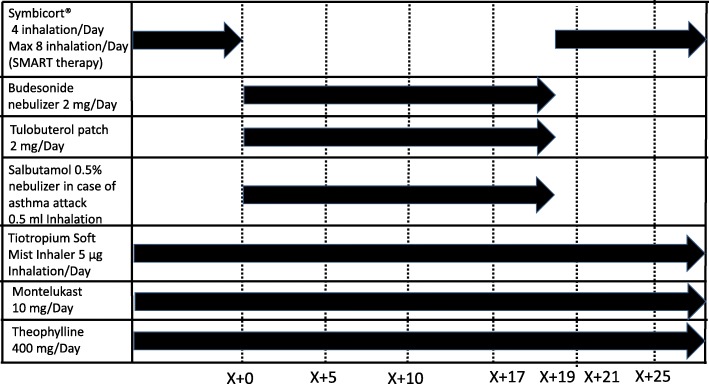
Prescription history in this case. X + 0 indicates the time of surgery with permanent tracheostomy. X + 17, 19, and 21 indicate the days from surgery to the start of respiratory distress, the consultation at the respiratory department of internal medicine, and discharge from the hospital, respectively

Based on these findings, nebulized budesonide 2000 μg and tulobuterol patch 2 mg were switched to 2 inhalations of Symbicort twice daily up to a total of 8 inhalations/day (SMART). The antiasthmatic drugs Spiriva Respimat and anti-IgE antibody, which had been added before surgery, were continued. The patient’s symptoms improved quickly over the course of 2 days after starting 2 inhalations of Symbicort in the morning and evening plus additional inhalations as needed (usually 1 inhalation in the evening). Owing to stabilized asthma symptoms, she was discharged on the third day after switching drugs (Fig. 1).

## Discussion

The efficacy of inhaled drugs after permanent tracheostomy has not been established. Although some case studies and small-scale studies have investigated their efficacy, mostly nebulizer-type inhaled drugs were used [1, 2]. Here, we report a patient with a tracheostomy whose asthma symptoms were improved by inhalation powder despite the concerns about an inadequate inspiratory flow rate to use a DPI. In this case, the patient first practiced using the Turbutester while simultaneously closing the tracheostomy with her hands, and then used the In-Check DIAL® to verify that the inspiratory flow rate was sufficient. After switching drugs, her asthma symptoms stabilized, showing that Symbicort inhalation powder was beneficial.

Comparison by component revealed that ICS and LABA were used both before and after switching drugs because nebulized budesonide (ICS) and tulobuterol patch (LABA) were used before switching, and Symbicort (ICS/LABA combination) was used after switching. However, previous studies of asthma and chronic obstructive pulmonary disease showed that ICS and LABA create a synergistic effect, suggesting that the efficacies of these drugs are higher when they are used concurrently [5, 6]. It has been shown that, compared with conventional therapy, SMART effectively suppresses acute exacerbations by increasing ICS when attacks occur [7]. It has also been reported that acute exacerbations are suppressed more effectively with medium-dose ICS in SMART than high-dose ICS in conventional therapy [8].

In this case, three factors are thought to have contributed to the improvement of asthma symptoms by Symbicort resumed after surgery: (i) synergetic effect provided by concurrent administration of ICS/LABA via the use of a drug containing both components [5], (ii) powerful bronchodilator action of formoterol [9, 10], and (iii) suppression of asthma exacerbations by SMART [7, 8].

Since various dry-powder inhalation and pMDI devices are available in Japan, it is not easy to find a more beneficial drug for the individual patient among these devices, including Symbicort [11, 12]. Drug distribution in the peripheral airways has been reported to differ between pMDI and DPIs; in particular, pMDI has smaller particle sizes compared with DPIs. Hence, inhalation via pMDI is thought to distribute the drugs into the peripheral airways more efficiently than DPIs [13]. On the other hand, when the same drugs are inhaled using different devices, some reports have indicated that the clinical effects are equivalent, while others suggest that there are some differences. Therefore, opinions on the relationship between clinical efficacy and the airway distribution of drugs are inconsistent [14, 15]. Moreover, it is not yet known which of the devices is the most appropriate or how much the drug distribution in the peripheral airways contributes to the clinical effects.

We tend to avoid the use of dry powder drugs for patients expecting to undergo a tracheostomy. However, because of large inter-individual variabilities in inspiratory flow rates and the skills required to use inhalation devices, it is important to carefully choose the most appropriate inhaled drug for each patient. It is difficult to gather enough information about patients with special conditions, like the patient in this case, and to verify the airway distribution of inhalation drugs. In addition, it is extremely difficult to detect subtle differences in airflow following tracheostomy. In the case of this patient, it is presumed that by closing the tracheostomy, respiration and drug distribution were almost the same as in patients without a tracheostomy.

## Conclusion

The use of Symbicort resulted in improved asthma control in a patient with severe asthma following a permanent tracheostomy. Thus, it is suggested that inhalation powders may be an option for patients with permanent tracheostomy.

## Abbreviations

DPI: Dry-powder inhaler
FEV1: Forced expiratory volume for 1 s
GINA: Global Initiative for Asthma
ICS: Inhaled corticosteroid
LABA: Long-acting β2 agonist
pMDI: Pressurized metered-dose inhaler
SABA: Short-acting β2 agonist
SMART: Single maintenance and reliever therapy
